# Complement system activation in wild boar (*Sus scrofa*) following parenteral administration of heat-inactivated *Mycobacterium bovis*

**DOI:** 10.3389/fvets.2025.1702063

**Published:** 2025-11-19

**Authors:** Margarita Villar, Oscar Rodríguez, Rita Vaz-Rodrigues, Angie E. Pardo-Reyes, Marta Rafael, Sara Artigas-Jerónimo, Gabriela de la Fuente, Isabel G. Fernández de Mera, Ramón A. Juste, Iker A. Sevilla, Lucas Domínguez, Christian Gortázar, José de la Fuente

**Affiliations:** 1 SaBio, Instituto de Investigación en Recursos Cinegéticos IREC-CSIC-UCLM-JCCM, Ciudad Real, Spain; 2 Biochemistry Section, Department of Inorganic, Organic Chemistry and Biochemistry, Faculty of Sciences and Chemical Technologies, Universidad de Castilla-La Mancha, Ciudad Real, Spain; 3 BP 30, Sidi Allal el Bahraoui, Morocco; 4 SabioTec S.L. Edificio Incubadora de empresas UCLM, Ciudad Real, Spain; 5 Biochemistry Section, Regional Center for Biomedical Research (CRIB), Faculty of Sciences and Chemical Technologies, University of Castilla-La Mancha, Ciudad Real, Spain; 6 Animal Health Department, NEIKER - Basque Institute for Agricultural Research and Development, Basque Research and Technology Alliance (BRTA), Derio, Bizkaia, Spain; 7 NySA, Servicio Regional de Investigación y Desarrollo Agroalimentario, Villaviciosa, Asturias, Spain; 8 VISAVET Health Surveillance Centre, Complutense University of Madrid, Madrid, Spain; 9 Department of Animal Health, Faculty of Veterinary Medicine, Complutense University of Madrid, Madrid, Spain; 10 Department of Veterinary Pathobiology, Center for Veterinary Health Sciences, Oklahoma State University, Stillwater, OK, United States

**Keywords:** complement, immunology, proteomic, tuberculosis, vaccine, wild boar

## Abstract

**Introduction:**

Development of vaccines to preserve and improve human and animal health requires effective protective antigens, delivery platforms, and adjuvants. The immunostimulant based on heat-inactivated *Mycobacterium bovis* (IV) was developed to boost protective immune response in different animal species against pathogen infection and tick infestations.

**Methods:**

In this study, a serum proteomics approach was used with functional annotations and enrichment network analysis for the characterization of immune pathways and biomarkers associated with parenteral administration of one, two, or three IV doses in the wild boar (*Sus scrofa*) animal model. An independent False Discovery Rate (FDR) analysis with the target-decoy approach provided by ProteinPilot™ was used, and positive identifications were considered when identified proteins reached a 1% FDR. Furthermore, pathogen surveillance was also performed to evaluate the IV treatment effect.

**Results:**

The proteomics analysis identified a total of 205 proteins, of which 97 displayed significant differential representation with 64 and 33 over (e.g., C4a, C5, C6, C7, and C9) and underrepresented (e.g., C3), respectively, in response to treatment. Results showed that IV administration activated both innate and adaptive immune responses through humoral immunity, regulation of the actin cytoskeleton pathway, coagulation cascade, and complement system. A single or two doses of IV significantly increased the activities of the classical, alternative, and lectin complement pathways. Moreover, a tendency was observed towards reducing seroprevalence in IV-treated wild boar over time for the causative agents of tuberculosis (*Mycobacterium tuberculosis* complex), pneumonia (*Mycoplasma hyopneumoniae*), and Aujeszky’s disease (porcine herpesvirus type 1).

**Discussion:**

These results support a role for IV in stimulating immune and anti-inflammatory responses with possible application in different vaccine formulations for the control of infectious diseases.

## 1 Introduction

From a One Health and sustainability perspective, vaccines are key interventions to preserve and improve human and animal health worldwide (1). For the development of effective, efficacious, and safe vaccines, protective antigens, delivery platforms and adjuvants are essential components (1–3). To advance understanding in vaccinology by addressing the development, formulation, production and evaluation of vaccines, an immunostimulant based on heat-inactivated *Mycobacterium bovis* (IV) was developed and demonstrated in different animal species such as cattle (*Bos taurus*), red deer (*Cervus elaphus*), badger (*Meles meles*), wild boar (*Sus scrofa*), pig (*Sus scrofa domesticus*), and zebrafish (*Danio rerio*) to boost specific protective immune responses against mycobacteria and non-specific cross-protective responses against other pathogens such as *Leishmania* and *Salmonella* species and ectoparasite tick vectors (4–9). For example, an oral vaccine formulation combining IV adjuvant with tick protective antigen Subolesin resulted in vaccine efficacy higher than 95% against *Rhipicephalus decoloratus* and *Rhipicephalus appendiculatus* infestations with negative tuberculin test, and thus not affecting tuberculosis diagnosis in cattle (8). These results support the possibility of using IV to advance in vaccinology (7, 10).

The levels of selected immune biomarkers in response to oral and/or parenteral IV formulations have been evaluated in different animal species (4, 6, 8, 9, 11–17) (Supplementary Table 1). In these experiments, the complement system is involved in innate immunity and linked with adaptive immunity (18, 19) and particularly complement component C3 upregulation was associated with protective effects in response to *Mycobacterium tuberculosis* complex (MTC) infection (12, 15) and against tick infestations (4).

The wild boar animal model has been validated in several proteomic studies focused on response to tuberculosis disease and/or IV protective response at the mandibular lymph node and oropharyngeal tonsil (20, 21), blood cells (14), secretome (22), microbiome [metaproteomics; (23)], and from genomics to proteomics comparative (24) levels.

Based on these results supporting the role of IV in immune response regulation, in this study, a serum proteomics approach was used for the first time to advance the characterization of immune pathways and biomarkers associated with prolonged parenteral IV treatment in the wild boar animal model. The study was focused on wild boar naturally exposed to pathogens affecting animal health with environmental implications under field conditions for the evaluation of immune mechanisms with possible protective capacity against multiple pathogens. The results support a role for IV in activating the complement system and other protective immune and anti-inflammatory responses with application in vaccine formulations. Furthermore, our findings sustain the activation of the innate immune system through different mechanisms, with differences between short-term (after one or two doses) and long-term (after three doses) responses to IV.

## 2 Materials and methods

### 2.1 Study site

The research was carried out in a private reserve with 3,000 ha, located in the Maamora Forest, Northwest Morocco (34°02′54.19′′ N, 6°27′19.24′′ W). This region presents low-elevation sandy soil (72–185 m above sea level) and has a Mediterranean bioclimate with hot and arid summers, an annual rainfall range of 300–500 mm with average annual temperature of 22 °C (25, 26). The dominant vegetation includes cork oak (*Quercus suber*) and a variety of endemic and Mediterranean species, such as wild pear (*Pyrus mamorensis*), wild olive (*Olea europaea*), and green olive (*Phylirea latifolia*). The reserve shows well-presented undergrowth species diversity and cover when compared to other regions in the forest that were overgrazed by livestock (27).

### 2.2 Experimental design

#### 2.2.1 Ethics statement

In the field trial, wild boars are maintained and treated with IV yearly, and sampling and analysis were approved by local ethical wildlife capture and management protocols (references B20/238-45/350-57 and B21/504-11/824-27/837-40). For the control pen trial (6), experimental design was in accordance with European (86/609) and Spanish laws (R.D. 223/1988, R.D. 1,021/2005), and the protocol was approved by the Committee on the Ethics of Animal Experiments of the Regional Agriculture Authority (Diputación Foral de Vizcaya, Permit Number: 27312009).

#### 2.2.2 Field trial experiment

**Figure 1:**
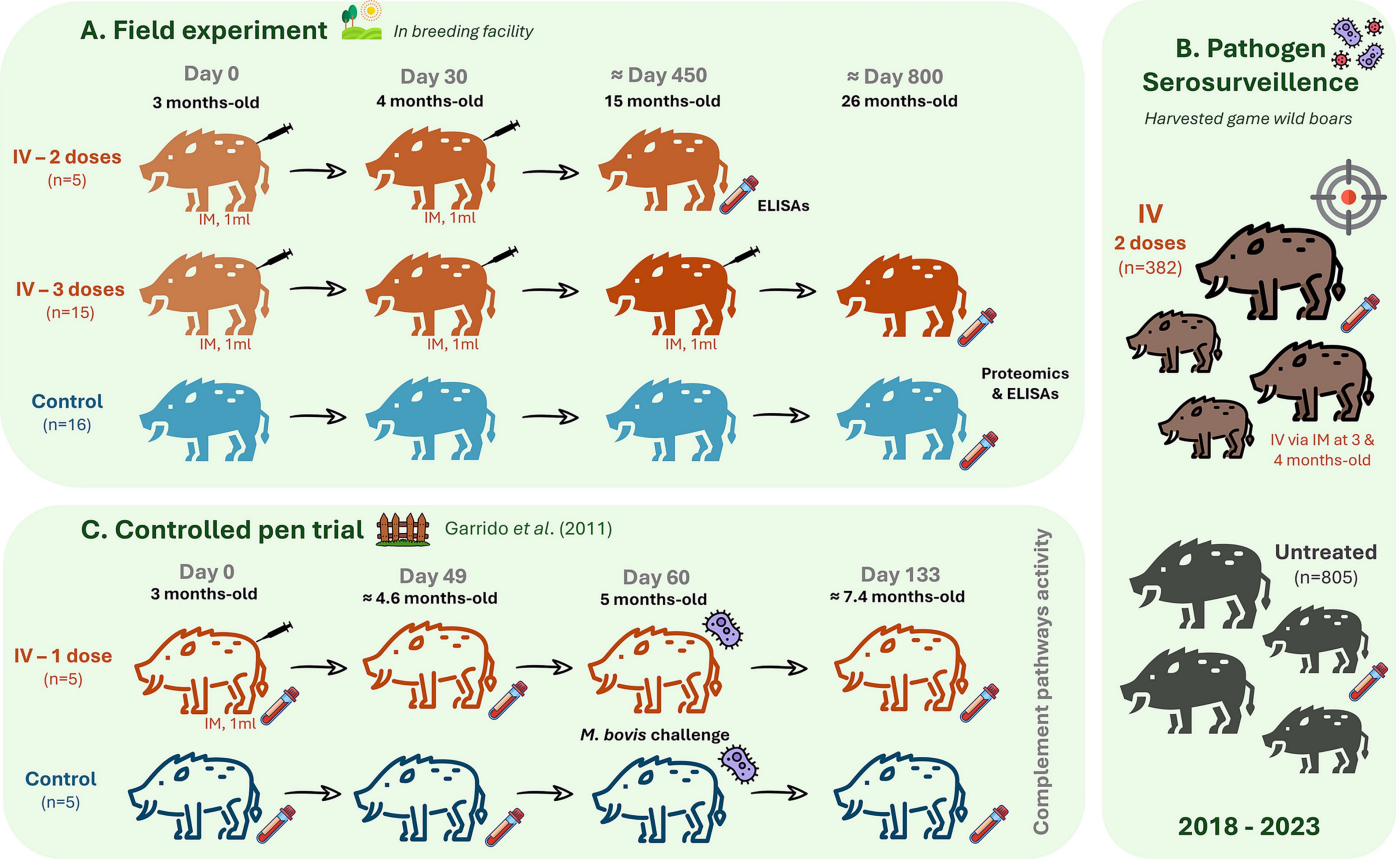
Experimental designs. **(A)** Field experiment presented three groups with randomly assigned wild boar piglets to a group that received via intramuscular (IM) two doses of 1 mL heat-inactivated *Mycobacterium bovis* (IV, 5 × 10^7^ cfu) at 3 and 4 months of age (*n* = 5 males), a group that was inoculated with three doses of IV at 3-, 4- and 15-months-old (*n* = 15, seven females and eight males) and an untreated control group (*n* = 16, eight females and eight males). Serum samples analysis included serum proteomics in animals treated with three doses of IV and controls (*n* = 5 per group). Analysis of serum IgG antibody against IV, biomarker protein levels, and complement activity with ELISAs was conducted in all animals. **(B)** Serosurveillance for pathogens was conducted in harvested game animals treated with mostly two doses of IV (*n* = 382) and untreated controls (*n* = 805) from 2018 to 2023. This analysis included the measurement of antibody titers against *Mycobacterium tuberculosis* complex (MTC), *Mycoplasma hyopneumoniae*, porcine herpesvirus type 1, and influenza A virus. **(C)** Controlled pen trial: retrieved from Garrido et al. (6) for analysis of classical, alternative, and lectin complement pathways activity in response to *Mycobacterium bovis* infection (5 mL with 10^6^ cfu via oropharyngeal) in control and IV-treated wild boar (*n* = 5 per group).

The study site is dedicated to recreational wild boar hunting, which has a breeding facility for restocking the hunting area. Due to natural exposure, the prevalence of tuberculosis in the wild boar population (15% in 2023, *n* = 123) and reduction after the beginning of treatment with IV in 2013 (35% in 2018, *n* = 184), the administration of IV is maintained yearly. For the field trial experimental design, 31 animals over 1.5-years-old from the breeding facility were selected for the study (Figure 1A). Five male wild boar piglets were treated with two IV doses at 3 and 4 months of age. Fifteen animals (seven females and eight males) were treated with three doses of IV at 3 months of age (first dose), 1 month later (4-months-old, second dose), and close to 1 year after the first dose (15-months-old, third dose). The IV was administered intramuscularly (IM) in piglets by inoculation of 1 mL IV (5 × 10^7^ cfu). Sixteen control animals of the same age and sex (eight females and eight males) were not treated. Blood samples were collected for analysis 11 months after the last dose and before the next dose administration of IV using sterile plastic tubes (Vacutainer®, Becton-Dickinson, NJ, USA) from the endocranial venous sinus (28). Blood was centrifuged at 4,000 × g for 15 min, and the obtained sera were stored at −20 °C until analysis. All serum samples collected in this experiment were used for the analysis of IgG antibodies against IV, complement activity, and biomarker protein levels via enzyme-linked immunosorbent assay (ELISA). Serum samples from immunized animals with three doses of IV and untreated controls were randomly selected for proteomics analysis (*n* = 5 males per group).

#### 2.2.3 Pathogen serosurvaillance in harvested game wild boars

For serosurveillance of pathogen prevalence, antibodies against *M. tuberculosis* complex (MTC), *Mycoplasma hyopneumoniae*, porcine herpesvirus type 1, and influenza A virus were measured. Sera were collected from hunted animals IV treated (most with two doses, *n* = 382) or not treated (*n* = 805) between 2018 and 2023 (Figure 1B). Additionally, IgG antibodies against the Crimean Congo Hemorrhagic Fever virus (CCHFV) were measured in all IV-treated animals (*n* = 20) and randomly selected untreated controls (*n* = 10). Ten ml blood were collected into a sterile plastic tube without additives (Vacutainer®, Becton-Dickinson, NJ, USA) via intracavernous venipuncture through bleeding from the cavernous sinus of the dura mater encephali (28) and centrifuged at 4,000 × g for 15 min. The extracted sera were stored at −20 °C until analysis.

#### 2.2.4 Controlled pen trial with IV administration and *Mycobacterium bovis* challenge

For analysis of complement pathways activity, additional serum samples were collected from wild boar (*n* = 5 per group) in a previous controlled experiment conducted by Garrido et al. (6) were included (Figure 1C). Briefly, 3–4-month-old piglets were randomly assigned to one of two groups: a treatment group that received 1 dose of 1 mL IV (6 × 10^6^ cfu) via Intramuscular (IM) at day 0 and an untreated control group. All animals were challenged with 5 mL containing 10^6^ cfu of an *M. bovis* field strain, administered by the oropharyngeal route at day 60, as described in previous experiments (6). Serum samples were collected from untreated controls before IV treatment (day 0), after treatment and before infection (day 49), and post-treatment plus challenge (day 133). Blood was collected via intracavernous venipuncture (28) into sterile plastic tubes (Vacutainer®, Becton-Dickinson, NJ, USA) and centrifuged at 4,000 × g for 15 min. The extracted sera were stored at −20 °C until analysis.

### 2.3 Production and formulation of IV

The *M. bovis* IV was produced as previously described (6, 12). Briefly, the *M. bovis* field isolate (strain Neiker 1,403; spoligotype SB0339) was cultured in Middlebrook 7H9 medium with OADC (10% v/v) supplement (Sigma-Aldrich, St. Louis, MO, USA), collected by centrifugation, washed, and resuspended in PBS and passed through an insulin syringe for declumping. The optical density of the suspension was adjusted with PBS to 5 McFarland units. Mycobacteria were inactivated in a shaking water bath at 81–83 °C for 40 min with a final IV preparation containing approximately the equivalent of 10^7^ cfu in 0.2 mL of sterile PBS.

### 2.4 Serum proteomics

Sera from the field experiment included five randomly selected males per group (three doses of IV and untreated controls) for proteomics analysis (Supplementary Data 1). Five male animals per group were selected for proteomics analysis, considering previous studies [e.g., (20, 24)], breeding conditions of the animals, which support low genetic diversity, and easier access to males due to higher investment of females in reproduction. Protein concentration in serum samples from immunized and control individuals was determined using the BCA Protein Assay (Bio-Rad, Hercules, CA, USA) with bovine serum albumin (BSA) dilutions as the standard. Protein serum samples (150 μg per sample) were trypsin digested using the FASP Protein Digestion Kit (Expedeon Ltd., UK) and sequencing grade trypsin (Promega, Madison, WI, USA) following the manufacturer’s recommendations. The resulting tryptic peptides were desalted onto OMIX Pipette tips C18 (Agilent Technologies, Santa Clara, CA, USA), dried down, and stored at −20 °C until mass spectrometry analysis. The desalted protein digests were resuspended (final concentration of 2 μg/μL) in 2% acetonitrile and 5% acetic acid in water and analyzed by reverse-phase liquid chromatography coupled online to mass spectrometry (RP-LC–MS/MS) using an Ekspert™ nLC 415 system coupled with a 6,600 TripleTOF mass spectrometer (AB Sciex, Framingham, MA, USA) through Information-Dependent Acquisition (IDA) followed by Sequential Windowed data-independent Acquisition of the Total High-Resolution Mass Spectra (SWATH-MS). The peptides were concentrated in a 0.1 × 20 mm C18 RP precolumn (Thermo Scientific, Waltham, MA, USA) with a flow rate of 5 μL/min for 10 min in solvent A. Then, peptides were separated in a 0.075 × 250 mm C18 RP column (New Objective, Woburn, MA, USA) with a flow rate of 300 nL/min. Peptide elution was done in a 60-min gradient from 5% to 30% solvent B, followed by a 15-min gradient from 30% to 60% solvent B (solvent A: 0.1% formic acid in water, solvent B: 0.1% formic acid in acetonitrile) and directly injected into the mass spectrometer for analysis. For IDA experiments, an equal number of the 10 samples for each vaccinated and non-vaccinated group were joined together as a representative mixed sample, which was used for the generation of the reference spectral ion library as part of SWATH-MS analysis. A total amount of 4 μg was injected in duplicate. The mass spectrometer was set to scan full spectra from 350 m/z to 1,400 m/z (250 ms accumulation time) followed by up to 50 MS/MS scans (100–1,500 m/z). Candidate ions with a charge state between +2 and +5 and counts per second above a minimum threshold of 100 were isolated for fragmentation. One MS/MS spectrum was collected for 100 ms, before adding those precursor ions to the exclusion list during 15 s (mass spectrometer operated by Analyst® TF 1.7, ABSciex®). Dynamic background subtraction was turned off. Data were acquired in high sensitivity mode with rolling collision energy on and a collision energy spread of 5. For SWATH quantitative analysis, 20 independent samples (two technical replicated from each of the five biological replicates for vaccinated and non-vaccinated groups) (4 μg each) were subjected to the cyclic Data Independent Acquisition (DIA) of mass spectra using the SWATH variable windows calculator (V 1.0, AB Sciex) and the SWATH acquisition method editor (AB Sciex) like previously established methods (29). A set of 50 overlapping windows was constructed (1 m/z for window overlap), covering the precursor mass range of 400–1,250 m/z, based on data from the IDA runs previously acquired. For these experiments, a 50-ms survey scan (350–1,400 m/z) was acquired at the beginning of each cycle, and SWATH-MS/MS spectra were collected from 100 to 1,500 m/z during 70 ms at high sensitivity mode, resulting in a cycle time of 3.6 s. Collision energy for each window was determined according to the calculation for a charge +2 ion-centered upon the window with a collision energy spread of 15. To create a spectral library of all detectable peptides in the samples, the IDA MS raw files were combined and subjected to database search in unison using ProteinPilot software v. 5.0.1 (AB Sciex) with the Paragon algorithm. Spectra identification was performed by searching against the Uniprot *Sus scrofa* proteome database (46,906 entries in September 2023) with the following parameters: iodoacetamide cysteine alkylation, trypsin digestion, identification focus on biological modification, and thorough ID as search effort. The detected protein threshold was set at 0.05. An independent False Discovery Rate (FDR) analysis with the target-decoy approach provided by ProteinPilot™ was used to assess the quality of identifications. Positive identifications were considered when identified proteins reached a 1% global FDR. The mass spectrometry proteomics data have been deposited to the ProteomeXchange Consortium via the PRIDE (30) partner repository with the dataset identifier PXD050002 and 10.6019/PXD050002.

### 2.5 Proteomics data analysis

For SWATH processing, the spectral alignment and targeted data extraction of DIA samples were performed using the SWATH Acquisition MicroApp 2.0 in the PeakView v. 2.2 software (ABSciex) with the reference spectral library. The parameters included up to 10 peptides per protein, seven transitions per peptide, 15 ppm ion library tolerance, 5 min XIC extraction window, 0.01 Da XIC width, and considering only peptides with at least 99% confidence and excluding those that were shared or contained modifications. However, to ensure reliable quantitation, only proteins with three or more peptides available for quantitation were selected for XIC peak area extraction and exported for analysis in the MarkerView v. 1.3 software (ABSciex). Global normalization was performed according to the Total Area Sums of all detected proteins in the samples.

The Student’s *t*-test (*p* < 0.001) was used to perform two-sample comparisons between the averaged area sums of all the transitions derived for each protein across the replicates for each group under comparison, to identify proteins that were significantly differentially represented between vaccinated and non-vaccinated individuals.

### 2.6 Functional annotations and enrichment analysis of proteomics data

The volcano plot highlighting significantly represented proteins of interest was created using the *EnhancedVolcano* package (31). To obtain the functional prolife of significant proteins, the gene ontology (GO) biological process (BP), molecular function (MF), and cellular component (CC) database was used at GO distribution level 3 with the *groupGO* function from the R package *clusterProfiler* (32–34). A further in-depth analysis of significant proteins was performed using over-representation analysis (ORA) based on Fisher’s exact test and applying the *weight01* method on the GO BP database (35). The gene set enrichment analysis (GSEA) was conducted using the GO database with the *gseGO* function from the *clusterProfiler* package (33, 34, 36), using 10,000 permutations and a Benjamini–Hochberg (BH) adjusted *p*-value cutoff of 0.05, in order to pinpoint significant pathways. Moreover, the Kyoto Encyclopedia of Genes and Genomes (KEGG) GSEA analysis was carried out with the *gseKEGG* function from the package *clusterProfiler* (33, 34, 36), employing 10,000 permutations and a BH-adjusted *p*-value lower than 0.05, to identify significant biochemical pathways. Genome-wide annotation for pig (*Sus scrofa*) was obtained from the mapping library of the R package *org.Ss.eg.db* (37). The R package *enrichplot* (38) was used for the visual representation of the functional enrichment results. The *Pathview* package was used for pathway-based data integration and visualization (39). A complementary network analysis was conducted using Metascape gene annotation and analysis resource^1^ for enriched ontology clusters (GO/KEGG), and a network of terms with a similar score >0.3 linked by an edge (40–42). The network on enriched ontology clusters was visualized with Cytoscape^2^ with “force-directed” layout. All complement components identified in wild boar by serum proteomics were analyzed in simplified pathways with protein representation differences in response to IV treatment.

### 2.7 Complement C3 analysis: Pearson correlation and multiple linear regression studies

Using data previously published about gene/protein differential biomarkers in response to IV and pathogen infection in different hosts (Supplementary Table 1), a Pearson correlation coefficient calculator (43) was used for C3 correlation analysis (*n* = 16, degrees of freedom = *n* − 2) (Supplementary Table 2). Multiple linear regression was also conducted using an online regression calculator (44) in order to identify C3 patterns of biological response to IV treatment and different pathogenic agents in several challenged species (Supplementary Table 2).

### 2.8 Humoral response to treatment with IV

Sera from all field trial experiment animals were used for analysis of IgG antibody levels to IV. Wild boar serum samples were tested through an in-house ELISA using IV as antigen, produced as abovementioned. ELISA plates were coated with 0.1 μg IV per well in carbonate–bicarbonate buffer (Sigma-Aldrich Inc., St. Louis, USA) and incubated overnight at 4 °C with gentle shaking. Then, plates were washed once with PBS containing 0.05% Tween-20 (PBST; Sigma-Aldrich, Munich, Germany), and subsequently blocked for 1 h with 2.5% skim milk powder (Condalab, Madrid, Spain) solution in PBS (block solution) at room temperature (RT). Serum samples were added in duplicate at a dilution of 1:100 in block solution and incubated for 1 h at 37 °C. Then, plates were washed three times with PBST, and goat anti-pig IgG HRP-conjugated (Bethyl Laboratories, Montgomery, USA) at a concentration of 1:10,000 in block solution was added and incubated for 1 h at RT with gentle agitation. Following three washes with PBST, 3,3′,5,5′ tetramethylbenzidine One Solution (TMB; Promega, Madison, USA) was added, and plates were incubated for 10 min in darkness at RT. The reaction was stopped with H_2_SO_4,_ and the optical density (O.D.) was measured at 450 nm (O.D._450 nm_). The results were determined by averaging each set of duplicate serum samples after subtraction from the averaged negative control uncoated wells. Antibody titers were expressed as the O.D._450 nm_ value and compared between IV-treated and control animals using a one-way ANOVA with the *post-hoc* Tukey’s Honestly Significant Difference (HSD) test (*p* < 0.05) with the R software, version 4.2.3 (32).

### 2.9 Analysis of serum biomarker protein levels and complement pathways activity

Sera from all field experiment animals were used for analysis of different biomarker levels and complement pathways activity by commercial pig ELISA tests, following the manufacturer’s instructions (Supplementary Data 2). Furthermore, serum samples from the controlled pen trial retrieved from Garrido et al. (6) were also included for the analysis of complement pathways activity. Results were compared between different groups by a one-way ANOVA with the *post-hoc* Tukey’s HSD test (*p* < 0.05) using the R software, version 4.2.3 (32).

- Protegrin-1 (NPG1) (MyBioSource, San Diego, USA) and complement component C7 (Abbexa Ltd., Cambridge, UK) assays. Serum samples were diluted in PBS at a concentration of 1:100 for NPG1 and 1:1000 for C7. Sera and kit standards were added in duplicate to the microtiter plate and incubated for 2 h at 37 °C. Following the incubation period, the liquid was discarded, and plates were incubated for 1 h at 37 °C with Detection Reagent A. Plates were then washed three times with kit washing buffer (WB), and Detection Reagent B was added and incubated for 1 h at 37 °C. Then, plates were washed five times with WB, and TMB substrate solution was added and incubated for 15 min at 37 °C in the dark. The stop solution was added, and absorbance at O.D._450 nm_ was determined. A standard curve was constructed with the absorbance of reference standard solutions and used to calculate serum protein concentrations.
- Pig complement pathways: classical, alternative, and lectin (Hycult Biotech, Uden, Netherlands). Serum samples were diluted in PBS at a concentration of 1:100 and incubated for 1 h at 37 °C. Plates were washed four times with WB, followed by the addition of diluted tracer solution and incubated for 1 h at 37 °C. Following four washes with WB, diluted streptavidin-peroxidase was added, and plates were incubated for another hour at 37 °C. Plates were washed four times with WB, TMB solution added, and plates were incubated for 30 min at RT in the dark. Then, the stop solution was added, and the absorbance at O.D._450 nm_ was determined. The percentage of complement pathway activity (CPA) was calculated with the mean absorbance of each set of duplicate serum samples (SS), positive (PC), and negative (NC) controls using the formula CPA (%) = [(SS-NC)/(PC-NC)] × 100.

### 2.10 Serosurveillance of pathogen prevalence

Analyses were conducted in wild boar serum samples collected between 2018 and 2023 for serosurveillance and animals from the field trial experiment (*n* = 20 for IV-treated and *n* = 10 for controls) (Supplementary Data 3). An in-house indirect P22 ELISA was carried out for IgG antibodies against *M. tuberculosis* complex (MTC) (tuberculosis, TB). Commercial ELISAs were performed to measure antibody titers against *Mycoplasma hyopneumoniae* (pneumonia), porcine herpesvirus type 1 (Aujeszky’s disease), and influenza A virus (swine flu). Additionally, serum IgG antibodies against the Crimean Congo Hemorrhagic Fever virus (CCHFV) were analyzed only in animals from the field experiment. The results were then used to evaluate IV treatment effect on pathogen infection (Supplementary Tables 3, 4).

For MTC, microplates were coated with 10 μg/mL *Mycobacterium* P22 protein complex antigen (45) in carbonate/bicarbonate buffer and incubated overnight at 4 °C. Wells were washed with 200 μL PBST. Plates were blocked for 1 h at RT with 100 μL/well of blocking buffer (PBS, 2.5% non-fat milk, pH 7.2) and washed thrice with 200 μL washing buffer. Then, 100 μL of wild boar serum diluted 1:100 in blocking buffer was added to the wells, and the plates were incubated at 37 °C for 1 h. Plates were washed as before, and 100 μL goat anti-pig IgG-Fc fragment-HRP conjugates diluted 1:10,000 in blocking buffer were added to the wells and incubated at RT for 1 h. Plates were washed as before, and 100 μL TMB was added to the wells and then incubated in the dark for 15 min at RT. Reactions were stopped by the addition of 50 μL of H_2_SO_4_ 3 N to measure O.D._450 nm_. Monoclonal specific antibodies against *M. hyopneumoniae* (INgezim M. hyo Compac, Ref. #11.MHYO.K3), pseudorabies virus (INgezim ADV gE PLUS, Ref. #11.GEP.K3), and influenza A virus (INgezim INFLUENZA PORCINA, Ref. #11.FLU.K1) were determined with commercial blocking or indirect ELISA kits (Ingenasa Gold Standard Diagnostics Companies, Budapest, Hungary), following the manufacturer’s instructions. CCHFV-specific IgG antibodies were detected using the IDScreen CCHF Double Antigen Multi-species commercial ELISA kit (IDVet, Grabels, France) and following the manufacturer’s instructions. Cut-off values for CCHFV sero-positive and sero-negative samples were determined according to the kit’s criteria.

## 3 Results

### 3.1 Proteome differential representation, functional annotation, and enrichment analysis

**Figure 2:**
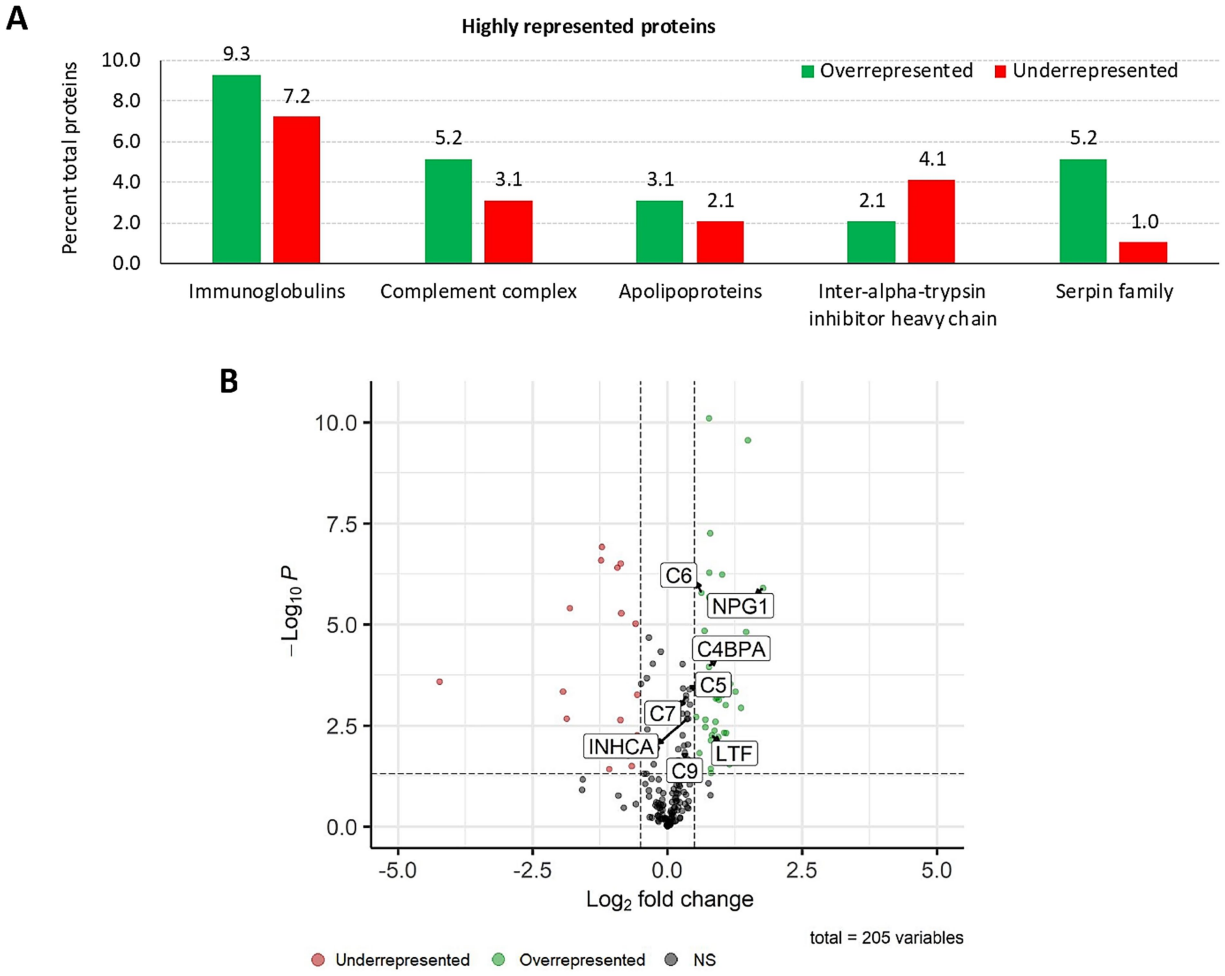
Proteomics: analysis of highly represented proteins in the wild boar serum proteome in response to IV. **(A)** Percent of total proteins for highly over and underrepresented proteins identified by serum proteomics analysis in response to IV treatment in the wild boar (*Sus scrofa*) animal model. **(B)** Graphic illustration of protein representation by means of a Volcano plot, highlighting significant proteins related to the activation of humoral immune response. Created with the *EnhancedVolcano* package using an adjusted *p*-value cutoff of 0.05 and a log_2_ fold change ≥ 0.5. The negative log10 FC *p*-value (−Log_10_*P*) is plotted in the *y*-axis. C4BPA, apolipoprotein R; C, complement component; INHCA, carbonic anhydrase inhibitor; IV, heat-inactivated *Mycobacterium bovis*; LTF, lactotransferrin; NPG1, pig protegrin-1; NS, not significant.

The SWATH-MS proteomics analysis enabled the identification of a total of 205 proteins, of which 97 displayed a significant differential representation (Figures 2A,B; Supplementary Data 1, sheets 1, 2). Highly represented proteins in response to IV were grouped into immunoglobulins, complement complex, apolipoproteins, inter-alpha-trypsin inhibitor heavy chain, and serpin family (Figure 2A). Among these differentially represented proteins, 64 were overrepresented while 33 were underrepresented in response to treatment. Of these, significantly represented proteins included complement components (C5, C6, C7, and C9) and apolipoprotein R (C4BPA) (Figure 2B). Additionally, keratin type II cytoskeletal 1, and keratin 75 were significantly overrepresented in response to IV. Functional analysis of GO pathways uncovered 66 BP, 21 MF, and 23 CC pathways associated with the overrepresented proteins. Underrepresented proteins were related to 27 BP, 7 MF, and 7 CC pathways (Supplementary Data 1, sheets 3, 4).

**Figure 3:**
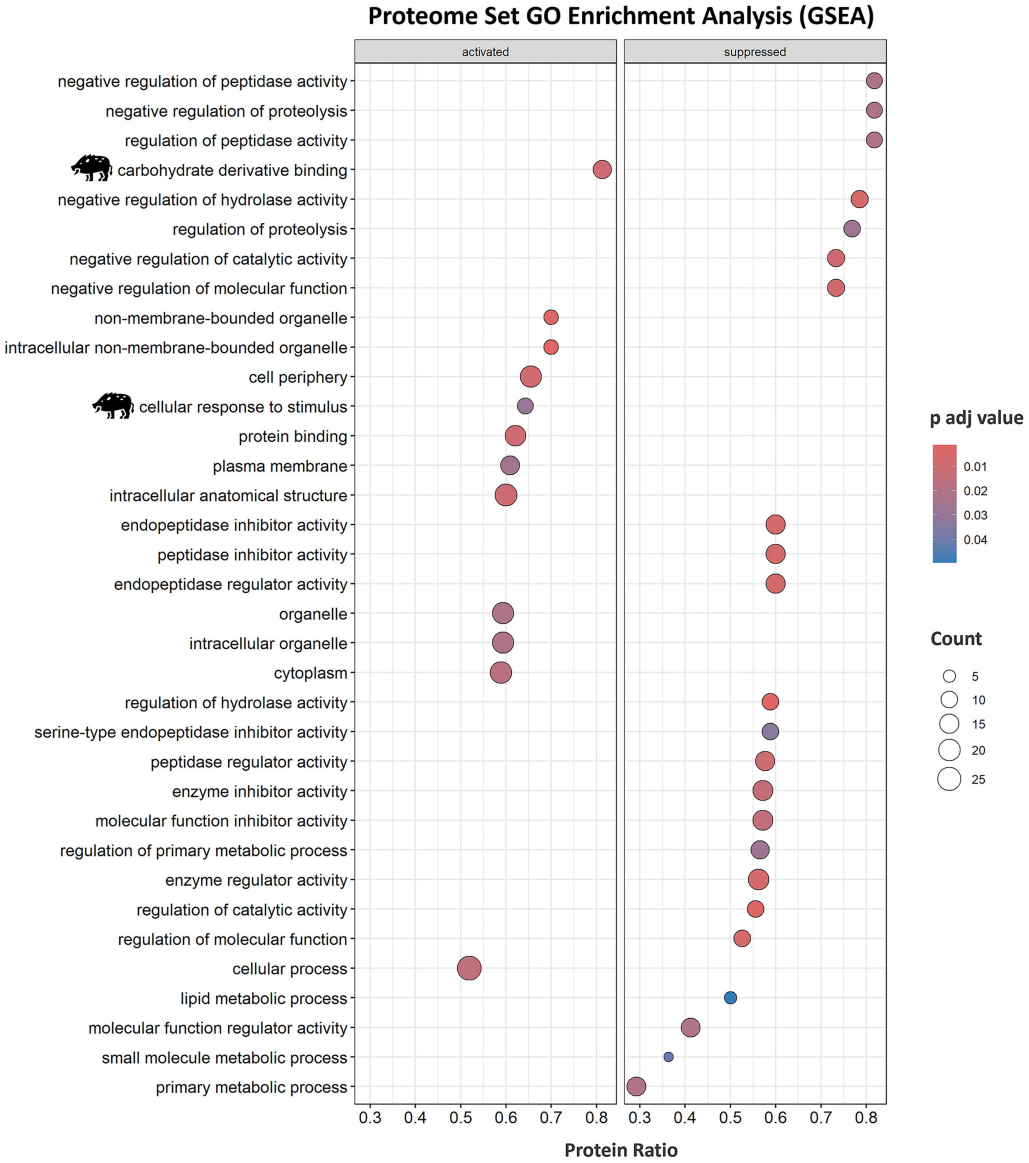
Serum proteome enrichment analysis of wild boar treated with the immunostimulant adjuvant based on IV. Proteome enrichment analysis was conducted using biological process (BP), molecular function (MF), and cellular component (CC) gene ontology (GO) database and executed with the *gseGO* function from *clusterProfiler* package using 10,000 permutations and a Benjamini–Hochberg (BH) adjusted *p*-value lower than 0.05. Significant pathways related to IV treatment are highlighted with a wild boar illustration. IV, heat-inactivated *Mycobacterium bovis*.

**Figure 4:**
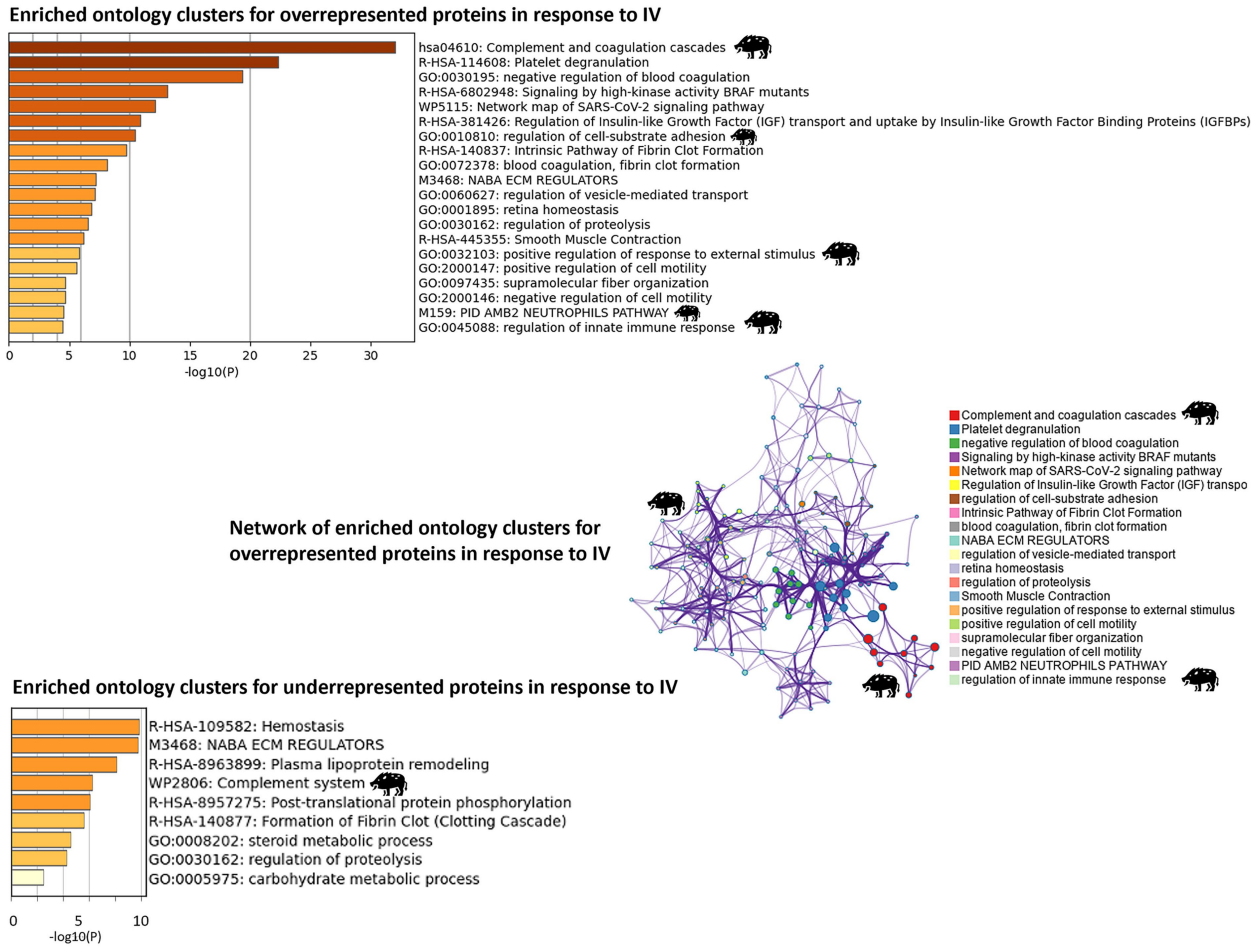
Proteomics: analysis of enriched ontology clusters and network. The analysis was conducted using Metascape (https://metascape.org) for enriched ontology clusters (GO/KEGG) for over and underrepresented proteins in response to heat-inactivated *Mycobacterium bovis* (IV) and a network of terms with a similar score >0.3 linked by an edge for overrepresented proteins (the thickness of the edge represents the similarity score). The network on enriched ontology clusters illustrates the functional relevance of biological pathways associated with overrepresented proteins, with the highest score for complement and coagulation cascades associated with IV treatment. The network was visualized with Cytoscape with a “force-directed” layout. Each significant cluster related to IV treatment is highlighted with a wild boar illustration.

In-depth enrichment analysis of significantly represented proteins revealed the activation of humoral immune response, which was linked to proteins lactotransferrin (LTF), inhibitor of carbonic anhydrase (INHCA), apolipoprotein R (APOR), protegrin 1, and complement components C5, C6, C7, and C9 (Figure 2B and Table 1). Activated pathways were also related to a positive regulation of response to stimulus and changes in the cellular component organization. Underrepresented proteins were associated with the suppression of the hydrolase activity regulation (Supplementary Data 1, sheets 5, 6). For all protein datasets, the GSEA method for GO BP, MF, and CC yielded a total of 35 significantly enriched pathways (12 overrepresented and 23 underrepresented) (Figure 3; Supplementary Data 1, sheet 7). Enriched ontology clusters and networks identified 20 and 9 clusters for over and underrepresented proteins, respectively (Figure 4). The ORA analysis showed humoral immune response, positive regulation of response to stimulus, and cellular component organization as significantly upregulated BP (Supplementary Data 1, sheet 5). Only the regulation of hydrolase activity was downregulated in response to IV (Supplementary Data 1, sheet 6). The enrichment analyses showed cellular response to stimulus and regulation of actin cytoskeleton as the most significantly overrepresented BP using GSEA (Supplementary Data 1, sheet 7) and GSEA KEGG (Supplementary Data 1, sheet 8) algorithms, respectively. Significant pathways related to IV treatment included carbohydrate derivative binding and cellular response to stimulus (Figure 3).

**Table 1:** Proteome functional annotation and enrichment analysis of immunologically relevant proteins in response to IV treatment.

Protein	Symbol	UniProt ID	Log_2_ FC	GO terms	Enrichment analysis (GO and KEGG; *p* < 0.05)
Protegrin-1	NPG1	A0A5G2QNY2	1.77	Defense response (GO:006952)	Humoral immune response and carbohydrate derivative binding
Lactotransferrin	LTF	Q6YT39	0.82	Regulation of cytokine production (GO:0001817)	Humoral immune response and cellular response to stimulus
Apolipoprotein R (Apo-R)	APOR or C4BPA	Q03472	0.77	T-cell mediated immunity (GO:0002456); negative regulation of complement activation, classical pathway (GO:0045959)	Humoral immune response, positive regulation of response to stimulus, and complement and coagulation cascades (NS)
Complement C6	C6	F1SMI8	0.63	Complement activation (GO:0006956)—classical pathway; innate immune response (GO:0045087)	Humoral immune response, positive regulation of response to stimulus, complement and coagulation cascades (NS), and regulation of actin cytoskeleton
Complement C5	C5	A0A287AIM8	0.40	Complement activation (GO:0006956)—classical and alternative pathways; chemokine production (GO:0032722)	Humoral immune response, positive regulation of response to stimulus, complement and coagulation cascades (NS), and regulation of actin cytoskeleton
Inhibitor of carbonic anhydrase	INHCA	I3LBF1	0.36	Extracellular region (GO:0005576)	Humoral immune response
Complement C7	C7	F1SMJ1	0.34	Complement activation (GO:0006956)—classical pathway; innate immune response (GO:0045087)	Humoral immune response, positive regulation of response to stimulus, complement and coagulation cascades (NS), and regulation of actin cytoskeleton
Complement C9	C9	A0A8W4F8A6	0.32	Complement activation (GO:0006956)—classical and alternative pathways; innate immune response (GO:0045087)	Humoral immune response, positive regulation of response to stimulus, complement and coagulation cascades (NS), and regulation of actin cytoskeleton

Immunologically relevant proteins are differentially overrepresented in the proteomics dataset. C, component; FC, fold change; GO: gene ontology; IV, heat-inactivated *Mycobacterium bovis*; KEGG, Kyoto Encyclopedia of Genes and Genomes; NS, not statistically significant.

The network on enriched ontology clusters highlighted complement and coagulation cascades and regulation of innate immune response associated with IV treatment (Figure 4). Similarly, the enrichment KEGG analysis revealed the significant activation of the regulation of actin cytoskeleton pathway and complement and coagulation cascades, with several proteins involved in these processes (Table 1; Supplementary Figure 1).

Other proteins of the complement system pathway such as C1r (A0A480RJC3, Log_2_FC = 0.13), C1q (C1q domain-containing proteins A0A287BRF1 and A0A286ZQJ9, Log_2_FC = −0.36 and −0.37; adiponectin Q6PP07, Log_2_FC = −0.86), C3 (F1S3H9, Log_2_FC = −0.57), and C4 (C4a Q03472, Log_2_FC = 0.77) involved in complement activation (GO:0006956) and innate immune response (GO:0045087) were differentially represented (*p* < 0.05) although without significant enriched pathways (Supplementary Data 1, sheets 1, 2). Complement components such as C2 (K7GPT9) and C8 alpha, beta, gamma chains (F6PYG1, F1S790, A0A287AFQ4, F1S788) were identified but without significant differences in response to IV treatment (*p* > 0.05) (Supplementary Data 1, sheets 1, 2).

### 3.2 Humoral response to IV

**Figure 5:**
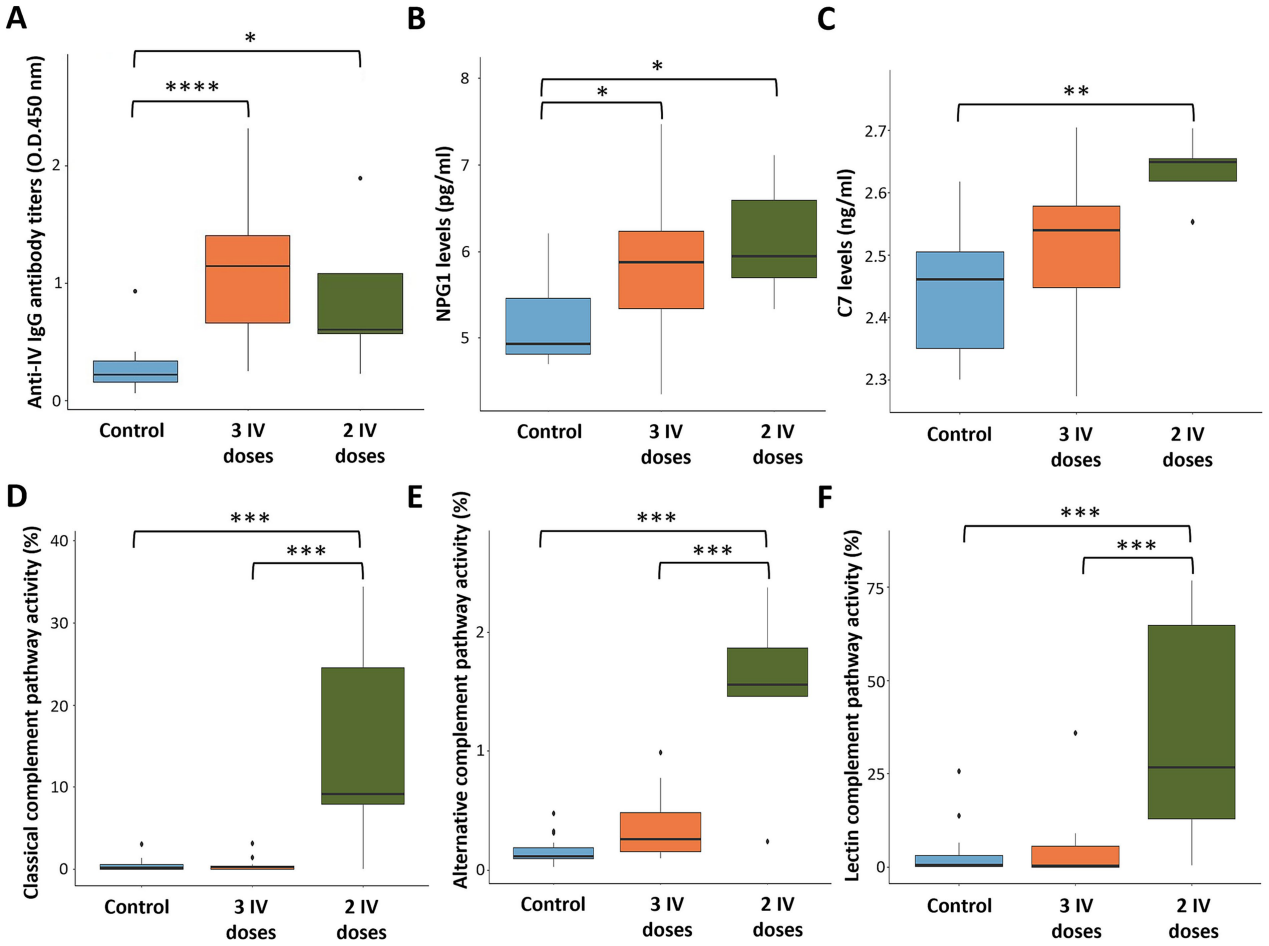
Serum biomarkers and antibody levels in response to heat-inactivated *Mycobacterium bovis* (IV). **(A)** Anti-IV IgG antibody levels. **(B)** NPG1 levels. **(C)** Complement C7 levels. **(D)** Classical complement pathway activity. **(E)** Alternative complement pathway activity. **(F)** Lectin complement pathway activity. ELISA O.D._450 nm_ values were compared between untreated (control) and treated with two and three doses of IV groups by a one-way ANOVA with the *post-hoc* Tukey’s Honestly Significant Difference (HSD) test using the R software, version 4.2.3 (32) (*p < 0.05, **p < 0.005, ***p < 0.0005, ****p < 0.00005; *n* = 5–15 animals per group).

Antibody response to IV was characterized in animals with 0, 2, and 3 IV treatments. The results showed significant differences (*p* < 0.05) in IV-treated animals when compared to untreated controls, without significant differences in IgG titers between IV treatments (Figure 5A).

### 3.3 Serum levels for selected biomarkers and complement pathway activity

For validation, serum levels of biomarkers NPG1 and C7 were analyzed by ELISA and compared to proteomics data (Supplementary Data 2). The results showed higher NPG1 levels in IV-treated animals with similar values after 2 and 3 IV doses (*p* < 0.05; Figure 5B). Notably, C7 serum levels were significantly elevated in animals treated with 2 IV doses (*p* < 0.005; Figure 5C), whereas a tendency toward higher levels was observed with 3 IV doses. Therefore, these results are consistent with the proteomics findings, which revealed higher serum concentrations for both NPG1 and C7 protein biomarkers in IV-treated animals (Supplementary Data 2).

**Figure 6:**
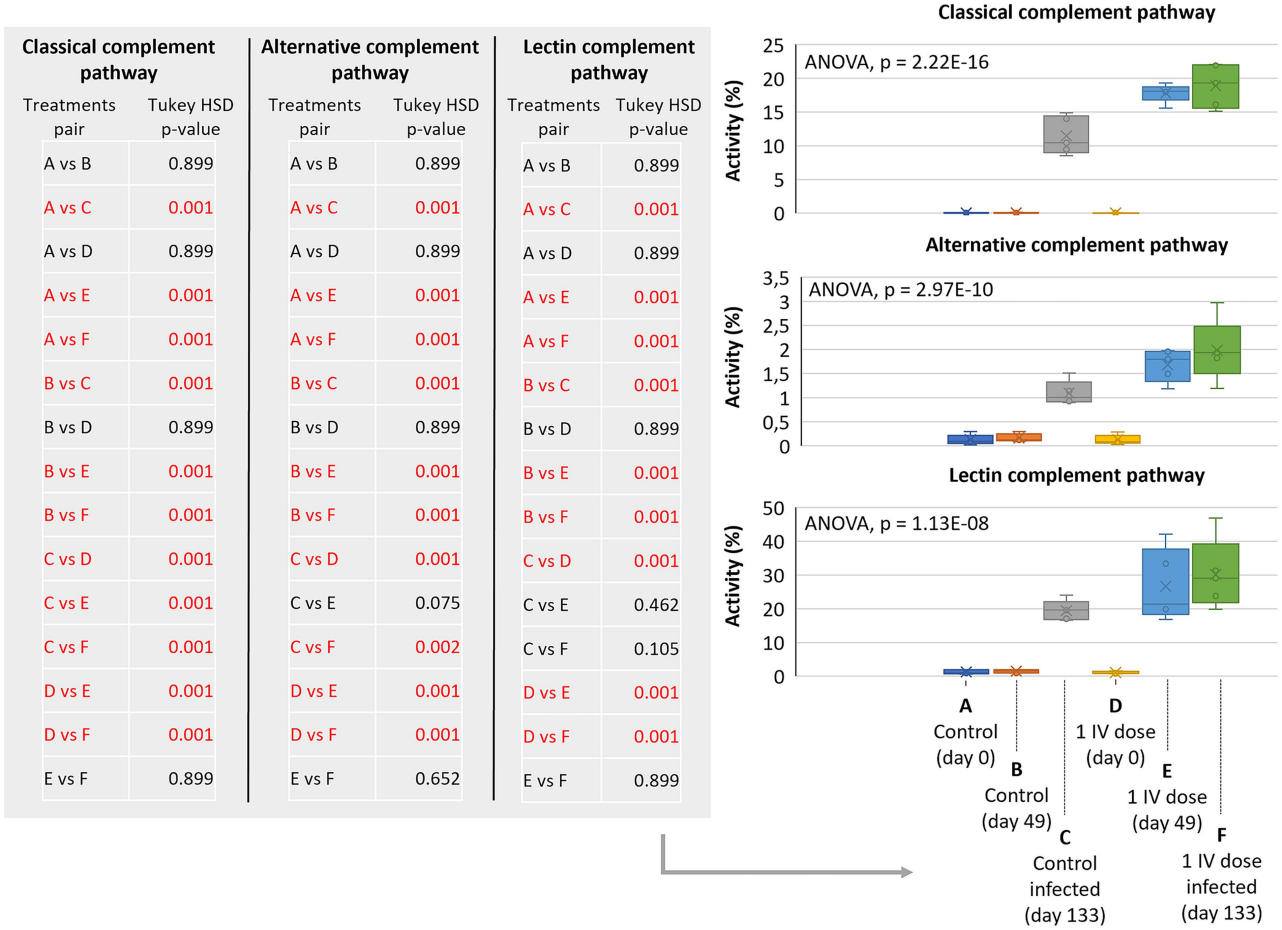
Complement pathways in response to IV treatment and *Mycobacterium bovis* infection in a controlled pen trial. Classical, alternative, and lectin complement pathways activity in response to *M. bovis* infection in control and 1 dose IV-treated wild boars. Serum samples were collected at days 0 (before treatment), 49 (before infection), and 133 (after infection) for analysis. ELISA O.D._450 nm_ values were compared between groups by a one-way ANOVA with the *post-hoc* Tukey’s Honestly Significant Difference (HSD) test using the R software, version 4.2.3 (32) (*p* = 0.001 highlighted in red for pair comparisons; *n* = 5 animals per group). IV, heat-inactivated *Mycobacterium bovis*.

**Figure 7:**
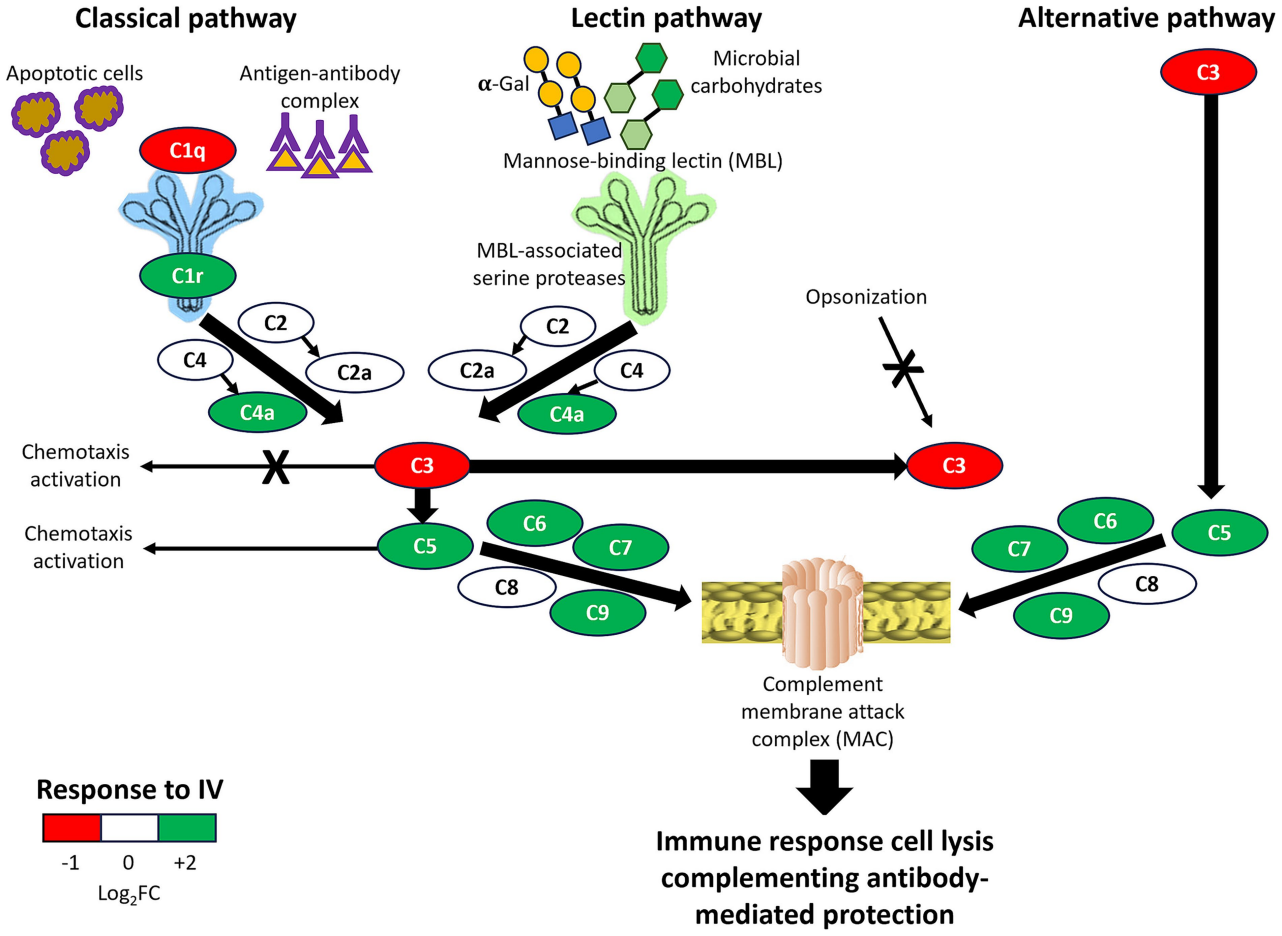
Overview of complement system pathways activity in response to IV. All complement components in classical, lectin, and alternative immune pathways were identified in wild boar serum proteomics analysis and shown in simplified pathways with representation differences in response to IV treatment (Log_2_ fold-change FC). Positive effects are shown with arrows, and negative effects by arrows with a cross. The final result by integrating all pathways is summarized as the immune response, cell lysis, and complementing antibody-mediated protection.

Classical, alternative, and lectin complement pathways were significantly activated in animals treated in this trial with 2 (*p* < 0.0005) but not 3 doses of IV and with high animal-to-animal variations (Figures 5D–F; Supplementary Data 2). To provide additional information on complement pathways in response to IV treatment and *M. bovis* infection, samples collected from a previous controlled pen trial (6) showed significant activation of classical, alternative, and lectin complement pathways in response to infection and IV treatment (*p* = 0.001; Figure 6). Furthermore, classical and alternative but not lectin complement pathways showed higher activity in IV-treated and infected wild boar when compared to infected controls at day 133 (p = 0.001; Figure 6). These results support activation of the complement system as part of the innate immune response to *M. bovis* infection and IV treatment, with differences between short- and long-term responses to IV (Figure 7).

### 3.4 Complement C3 correlation analysis in response to IV in different host species

Pearson correlation and multiple regression analyses with data from previous studies of IV treatment in different hosts (Supplementary Table 1) showed no significant differences in C3 regulation in response to oral IV administration in cattle (*Bos taurus*) and zebrafish (*Danio rerio*) or in response to *Mycobacterium* infection (Supplementary Table 2). These results agree with the C3 underrepresentation identified by serum proteomics analysis in wild boar treated with parenteral IV and sampled after the third treatment, 1 year after the first dose.

### 3.5 Pathogen seroprevalence in wild boar

Based on data of pathogen seroprevalence, wild boars were not highly exposed to *M. tuberculosis* complex (MTC), *Mycoplasma hyopneumoniae*, porcine herpesvirus type 1 (pseudorabies virus), and CCHFV (Supplementary Table 3). Only the seroprevalence of influenza A virus was higher than 20% in treated and control animals (Supplementary Table 3). However, the percentage of seroprevalence varied over time and between pathogens, which translated into differences in IV treatment with negative (−18.9% to − ∞%) and positive (85.7–100%) values (Supplementary Table 4). Nevertheless, except for the influenza A virus, a tendency was observed towards decreasing seroprevalence for different pathogens over time in IV-treated wild boar (*R*^2^ ranging from 0.26 to 0.42; Supplementary Table 4). Most of the animals from the field trial experiment were negative for the detection of pathogen infection (Supplementary Data 3), thus providing little impact on this analysis.

## 4 Discussion

The results of serum proteomics analysis support the activation of innate and adaptive immune responses through the humoral immune response, complement system, and innate immunity in response to parenteral IV treatment. The complement system plays a role in both innate and adaptive immunity for modulating host-pathogen interactions and contains proteins with the capacity for therapeutics and biomarkers in response to vaccination (18, 46–49). Complement C1q is the first component of the classical pathway, which binds to antibody–antigen complexes and also directly to some pathogens and apoptotic cells, with an influence on macrophage inflammatory responses (18, 50). Although C1q was downregulated in response to IV (Figure 7), the classical complement pathway was activated with a balance between protective immune and anti-inflammatory responses (18, 50). A balance between different complement system pathways (e.g., activation of C5-mediated classical pathway and downregulation of C3-mediated alternative pathway; Figure 7) results in activation of antimicrobial responses while reducing risks for inflammatory diseases and pathogen-associated immunothrombosis (18). For example, vaccination with the Bacille Calmette-Guérin (BCG) vaccine against tuberculosis has been associated with a higher risk of the pandemic of coronavirus disease 19 (COVID-19) likely associated with inflammatory reactions in response to BCG activation of certain innate immune mechanisms (51, 52). Accordingly, most complement therapeutics under clinical development target C3 and C5 components (18).

Trained immunity is associated with innate immune response memory driven by epigenetic reprogramming of innate immune cells to enhance their defense capabilities against secondary infections (53) but can also provoke inflammatory reactions and autoimmunity (7, 54, 55). Treatment with IV or exposure to alpha-Gal-containing biomolecules present in IV (56) have been shown to induce anti-inflammatory and trained immunity protective responses against pathogen infection, in which complement system pathways may be involved (7, 56–59). These results agree with activation of complement pathways in response to *M. bovis* infection and treatment with IV, with an effect on infection control, and with lectin pathway regulation, probably associated with reduced inflammatory reactions linked to C3 activation (57). The inflammatory response BP was evaluated with proteomics data and identified without significant differences (*p* = 0.352, Supplementary Data 1). Although inflammasome activation has a positive effect on triggering the innate immune response, it is also associated with negative effects contributing to cardiovascular and neurodegenerative disorders, among others (58), thus suggesting a positive effect of IV treatment in wild boar.

The differences in IV treatment for different pathogens may be related to temporal and pathogen/host genetic factors affecting pathogen transmission and host immunity. For example, it has been shown that pseudorabies virus affects host innate immunity through different mechanisms, including binding of virus glycoprotein III to C3 derived from the porcine natural host but not other species, to reduce complement activation and protective immune response to virus infection (60, 61). Additionally, it has also been shown that the keratinization pathway is significantly enriched in cattle with multifocal lesions in response to paratuberculosis (62). Nevertheless, the results showed pathogen seroprevalence without clinical signs and mortality, thus supporting an increased protective response to IV after annually repeated treatments.

Another consideration is the possible establishment of latent/chronic infections with risks associated with pathogen prevalence and transmission from reservoir hosts. This effect has been associated with tuberculosis and BCG vaccination (63, 64). Regarding IV, it has been shown that protective immune responses not only against mycobacteria but also non-specific cross-protective responses against other pathogens and ectoparasite vectors (4–9). Therefore, although supported by proteomics results shown here, which are still to be proven, the activation of multiple immune mechanisms in response to IV may reduce the risks for establishing latent/chronic infections. The cross-pathogen protective mechanisms associated with IV treatment may be regulated by trained immunity (TRAIM), defined as immune memory in which innate immune cells, such as monocytes, macrophages, dendritic, and natural killer (NK) cells, undergo an epigenetic reprogramming with enhanced primary protective capacity mediated by complement pathways, among other mechanisms (7).

## 5 Conclusion

In conclusion, the results showed differences between short- and long-term responses to IV through activation of different components of the innate immune system in classical, lectin, and alternative complement pathways, resulting in the formation of the complement membrane attack complex (MAC). As an effector of the immune system, the MAC is the final stage of the complement system terminal pathway that induces immune response cell lysis, complementing antibody-mediated adaptive immunity (65) (Figure 7). The MAC-associated mechanisms have a protective capacity against multiple pathogens with putative capacity to reduce risks associated with latent/chronic infections. A possible tolerization after three IV doses mediated, for example, by a decrease in C7 levels suggested the possibility of using a combination of these biomarkers (maximum non-specific/minimum specific responses) as a TRAIM test. However, further validation is required for the long-term tolerization hypothesis in response to IV.

The results of the study have potential implications in vaccine development for disease prevention and control. The role of IV in stimulating protective immune and anti-inflammatory responses has possible applications in different vaccine formulations for the control of infectious diseases. The IV can be used as an immunostimulant in vaccine formulations to boost non-pathogen-specific protective immune response [e.g., (8)]. Future directions should consider the use of IV alone and in combination with protective antigens and probiotic bacteria in oral and injected vaccine formulations.

## Data Availability

Data associated with this study are included in the paper and its additional information. Proteomics data was deposited in the ProteomeXchange Consortium via the PRIDE (30) partner repository with the dataset identifier PXD050002 and http://central.proteomexchange.org/cgi/GetDataset?ID=PXD050002.

## Footnotes

^1^ https://metascape.org/gp/index.html#/main/step1

^2^ https://cytoscape.org

## Supplementary material

SUPPLEMENTARY TABLE 1 Previous reports of gene/protein differential biomarkers in response to IV treatment and pathogen infection in different hosts.

SUPPLEMENTARY TABLE 2 Data for Pearson correlation analysis.

SUPPLEMENTARY TABLE 3 Data for pathogen seroprevalence in IV-treated and control non-treated wild boar.

SUPPLEMENTARY TABLE 4 Pathogen seroprevalence per group (IV vs. control) over time (2018–2023).

SUPPLEMENTARY DATA SHEET 1 Proteomics analysis.

SUPPLEMENTARY DATA SHEET 2 Serum biomarkers.

SUPPLEMENTARY DATA SHEET 3 Serosurveillance of pathogen prevalence.

SUPPLEMENTARY FIGURE 1 Complement and coagulation cascades pathways.
