# Supplementary material for: Complement system activation in wild boar (Sus scrofa) following parenteral administration of heat-inactivated Mycobacterium bovis
Source: Front Vet Sci. 2025 Nov 19;12:1702063. doi: 10.3389/fvets.2025.1702063 (PMC12673616; doi:10.3389/fvets.2025.1702063)
Supplement: SUPPLEMENTARY TABLE 4 — Pathogen seroprevalence per group (IV vs. control) over time (2018–2023). [file Supplementary_file_1.pdf]

Supplementary information

Supplementary Table 1. Previous reports of gene/protein differential biomarkers in response to IV treatment and pathogen infection in different hosts.

**Treatment formulation /doses**	**Host/Challenge**	**Sample/ days after 1st dose**	**Biomarkers in response to treatment**		**Reference**
			**Overrepresented**	**Underrepresented**	
Oral IV+SUB/2	Cattle/ *Rhipicephalus microplus*	Blood/65	*C3, IL-1β, IL-2, IL-12, akr2*	None	Contreras et al., 2019
Oral IV			*IL-2*	None	
Parenteral IV/2	Zebrafish/ *Mycobacterium marinum*	Peritoneal cavity organs/ 56-91	*C3, IL-1β, akr2*	None	López et al., 2018a
Oral (O)/2 or parenteral (P) IV/2	Zebrafish/ *Mycobacterium marinum*	Intestine/ 56	None	None	Ferreras-Colino et al., 2024
Oral (O)/2 or parenteral (P) BCG/2			*TLR2, TLR4* (P)	None	
Oral IV/1	Wild boar/ *Mycobacterium bovis*	PBMC/60	*MUT*	None	Garrido et al., 2011
Parenteral IV/1		Blood/60	IFN-γ	None	
Oral IV/2	Wild boar/ *Mycobacterium bovis*	PBMC/ 126	*C3, IL-1β, TRIF, NLRP3, MYD88, MUT*	None	Beltrán-Beck et al., 2014
		Serum/ 126	IL-1β, IL-6, IFN-γ		
Oral IV/2	Zebrafish/ *Mycobacterium marinum*	Peritoneal cavity organs/56	*C3, IL-1β*	None	Risalde et al., 2018
Parenteral IV/2	Cattle/ *Mycobacterium bovis*	Blood/84	C8b, TLR-2, TLR-9	C8a, IL1RL2	López et al., 2018b
Oral IV/2	Pig/*Salmonella enterica* serovar Choleraesuis	Lung Serum/28	*CCL28* TNF-α	None	Vaz-Rodrigues et al., 2022
Oral IV/2	Red deer/ *Mycobacterium bovis*	Serum/ 90-120	TNF-α	IL-10	Thomas et al., 2017
Oral BCG/2			None	IL-10	
Oral IV+SUB/2	Cattle/ *Rhipicephalus decoloratus Rhipicephalus appendiculatus*	Serum/ 140	IL-1β, TNF-α	None	Kasaija et al., 2022
Oral IV/2			None	None	

Gene mRNA biomarkers are shown in italics. Abbreviations: Complement component 3 (C3), Interferon gamma (IFN-γ), Interleukin (IL), Methylmalonyl-CoA mutase (MUT), Toll-like receptor adaptor molecule 1 (TRIF), NLR family, pyrin domain containing 3 (NLRP3), Myeloid differentiation primary response protein 88 (MYD88), Tumor necrosis factor alpha (TNF-α), Complement C8 alpha chain (C8a), Complement C8 beta chain (C8b), Toll-like receptor 2 (TLR-2), Toll-like receptor 9 (TLR-9), Interleukin 1 receptor like 2 (IL1RL2), peripheral blood mononuclear cells (PBMC).

References

Beltrán-Beck B, de la Fuente J, Garrido JM, Aranaz A, Sevilla I, Villar M, Boadella M, Galindo RC, Pérez de la Lastra JM, Moreno-Cid JA, Fernández de Mera IG, Alberdi P, Santos G, Ballesteros C, Lyashchenko KP, Minguijón E, Romero B, de Juan L, Domínguez L, Juste R, Gortazar C. Oral vaccination with heat inactivated *Mycobacterium bovis* activates the complement system to protect against tuberculosis. PLoS One. 2014;9(5):e98048. doi: 10.1371/journal.pone.0098048

Contreras M, Kasaija PD, Merino O, de la Cruz-Hernandez NI, Gortazar C, de la Fuente J. Oral Vaccination With a Formulation Combining *Rhipicephalus microplus* Subolesin With Heat Inactivated *Mycobacterium bovis* Reduces Tick Infestations in Cattle. Front Cell Infect Microbiol. 2019;9:45. doi: 10.3389/fcimb.2019.00045

Ferreras-Colino E, Contreras M, Risalde MA, et al. Heat-inactivated mycobacteria activate the toll-like receptor 2 and 4 pathways in the zebrafish model of tuberculosis. Vaccine. 2024. doi:10.1016/j.vaccine.2023.12.085

Garrido JM, Sevilla IA, Beltrán-Beck B, Minguijón E, Ballesteros C, Galindo RC, Boadella M, Lyashchenko KP, Romero B, Geijo MV, Ruiz-Fons F, Aranaz A, Juste RA, Vicente J, de la Fuente J, Gortázar C. Protection against tuberculosis in Eurasian wild boar vaccinated with heat-inactivated *Mycobacterium bovis*. PLoS One. 2011;6(9):e24905. doi: 10.1371/journal.pone.0024905

Kasaija PD, Contreras M, Kabi F, Mugerwa S, Garrido JM, Gortazar C, de la Fuente J. Oral vaccine formulation combining tick Subolesin with heat inactivated mycobacteria provides control of cross-species cattle tick infestations. Vaccine. 2022;40(32):4564-4573. doi: 10.1016/j.vaccine.2022.06.036

López V, Risalde MA, Contreras M, Mateos-Hernández L, Vicente J, Gortázar C, de la Fuente J. Heat-inactivated *Mycobacterium bovis* protects zebrafish against mycobacteriosis. J Fish Dis. 2018a;41(10):1515-1528. doi: 10.1111/jfd.12847

López V, van der Heijden E, Villar M, Michel A, Alberdi P, Gortázar C, Rutten V, de la Fuente J. Comparative proteomics identified immune response proteins involved in response to vaccination with heat-inactivated *Mycobacterium bovis* and mycobacterial challenge in cattle. Vet Immunol Immunopathol. 2018b;206:54-64. doi: 10.1016/j.vetimm.2018.10.013

Risalde MA, López V, Contreras M, Mateos-Hernández L, Gortázar C, de la Fuente J. Control of mycobacteriosis in zebrafish (*Danio rerio*) mucosally vaccinated with heat-inactivated *Mycobacterium bovis*. Vaccine. 2018;36(30):4447-4453. doi: 10.1016/j.vaccine.2018.06.042

Thomas, J., Risalde, M. Á., Serrano, M., Sevilla, I., Geijo, M., Ortíz, J. A., Fuertes, M., Ruíz-Fons, J. F., de la Fuente, J., Domínguez, L., Juste, R., Garrido, J., Gortázar, C. The response of red deer to oral administration of heat-inactivated *Mycobacterium bovis* and challenge with a field strain. Vet Microbiol. 2017;208:195–202. https://doi.org/10.1016/j.vetmic.2017.08.007

Vaz-Rodrigues R, Ferreras-Colino E, Ugarte-Ruíz M, Pesciaroli M, Thomas J, García-Seco T, Sevilla IA, Pérez-Sancho M, Mateo R, Domínguez L, Gortazar C, Risalde MA. Nonspecific protection of heat-inactivated *Mycobacterium bovis* against *Salmonella choleraesuis* infection in pigs. Vet Res. 2022;53(1):31. doi: 10.1186/s13567-022-01047-8

Supplementary Table 2. Data for Pearson correlation analysis.

Nominal 1	Nominal 2	Nominal 3	Nominal 4	Metric 1	Metric 2	Metric 3
**Formulation**	**Host**	**Challenge**	**Tissue**	**Sample time**	**C3 up**	**C3 down**
Oral IV-SUB	Cattle	Tick	Blood	65	1	0
Oral IV	Cattle	Tick	Blood	65	0	0
Parenteral IV	Zebrafish	Mycobacterium	Intestine	56	1	0
Oral IV	Zebrafish	Mycobacterium	Intestine	56	0, ND	0, ND
Parenteral IV	Zebrafish	Mycobacterium	Intestine	56	0, ND	0, ND
Oral IV	Wild boar	Mycobacterium	Blood	60	0	0
Parenteral IV	Wild boar	Mycobacterium	Blood	60	0	0
Oral IV	Wild boar	Mycobacterium	Blood	126	1	0
Oral IV	Wild boar	Mycobacterium	Serum	126	0	0
Oral IV	Zebrafish	Mycobacterium	Intestine	56	1	0
Parenteral IV	Cattle	Mycobacterium	Blood	84	0, ND	0, ND
Oral IV	Pig	Salmonella	Serum	28	0, ND	0, ND
Oral IV	Red deer	Mycobacterium	Serum	120	0	0
Oral BCG	Red deer	Mycobacterium	Serum	120	0	0
Oral IV-SUB	Cattle	Tick	Serum	140	0	0
Oral IV	Cattle	Tick	Serum	140	0	0

Data derive from Supplementary Table 1. Oral IV = 0, Oral IV-SUB = 1, Parenteral IV = 2, Oral IV-BCG = 3. Cattle = 1, Zebrafish = 2, Wild boar = 3, Pig = 4, Red deer = 5. Salmonella = 0, Tick = 1, Mycobacterium = 2. Blood = 1, Intestine = 2, Serum = 3. Abbreviation: ND, not determined. Pearson correlation coefficient calculator (https://www.socscistatistics.com/tests/pearson/) was used for analysis (n = 16, degrees of freedom = n - 2). Multiple linear regression was conducted with Statistics Kingdom (https://www.statskingdom.com/410multi_linear_regression.html).

Pearson correlation coefficient

Nominal 1 vs. Metric 2, r (14) = -0.0356, p = 0.89.
Nominal 2 vs. Metric 2, (a) r (14) = -0.1912, p = 0.47.
Nominal 3 vs. Metric 2, r (14) = 0.1204, p = 0.66.
Nominal 4 vs. Metric 2, r (14) = -0.3333, p = 0.21.
Metric 1 vs. Metric 2, r (14) = -0.1471, p = 0.59.

Results for both including or excluding ND entries:
Not significant C3 upregulation in response to Oral IV.
Not significant C3 upregulation in cattle and zebrafish.
Not significant C3 upregulation in response to Mycobacterium infection.
Not significant C3 upregulation in blood samples.
Not significant C3 upregulation closer to first immunization.

Multiple linear regression

X1 = Nominal 1 (Oral IV-SUB, Oral IV, Parenteral IV), X2 = Nominal 2 (Cattle, Zebrafish, Wild boar), X3 = Nominal 3 (Salmonella, Tick, Mycobacterium), Y = Metric 2 (C3 up or not). Very strong collective non-significant effect between X1, X2, X3 and Y [$F(3, -1) = -0.96$, $p = 0.50$, $r^2 = 0.74$, $r^2_{adj} = 1.52$].

Supplementary Table 3. Data for pathogen seroprevalence in IV-treated and control nontreated wild boar.

**IV treatment**	**TB (%)**		**Pneumonia (%)**		**Pseudorabies (%)**		**Flu (%)**	
	YES	NO	YES	NO	YES	NO	YES	NO
Treated (n = 382)	14.1	85.9	2.9	97.1	1.0	99.0	42.7	57.3
Not treated (n = 805)	5.8	94.2	6.3	93.7	0.4	99.6	21.6	78.4

Analyses were conducted by ELISA for *Mycobacterium* P22 (tuberculosis, TB), *Mycoplasma hyopneumoniae* (pneumonia), Aujeszky's disease (pseudorabies), influenza A virus (flu) and Crimean Congo Hemorrhagic Fever virus CCHFV (CCHF). Materials and methods are described in the manuscript. For CCHFV all animals tested negative (Supplementary Data 3).

Supplementary Table 4. Pathogen seroprevalence per group (IV treated vs. untreated control) over time (2018-2023).

Year	IV treated (n)	TB (n) YES	TB (n) NO	Pneumonia (n) YES	Pneumonia (n) NO	Pseudorabies (n) YES	Pseudorabies (n) NO	Flu (n) YES	Flu (n) NO
**2018**									
93	YES	43	50	6	87	4	89	78	15
91	NO	22	69	41	50	2	89	33	58
**E (%)**		-91.3		85.7		-95.7		-131.3	
**2019**									
67	YES	2	65	4	63	0	67	1	66
261	NO	0	261	3	258	0	261	46	215
**E (%)**		-oo		-419.4		---		91.5	
**2020**									
99	YES	7	92	0	99	0	99	18	81
85	NO	1	84	1	84	1	84	13	72
**E (%)**		-501.0		100.0		100.0		-18.9	
**2021**									
79	YES	1	78	1	78	0	79	38	41
105	NO	1	104	1	104	0	105	15	90
**E (%)**		-32.9		-32.9		---		-236.7	
**2022**									
14	YES	1	13	0	14	0	14	0	14
170	NO	4	166	0	170	0	170	12	158
**E (%)**		-203.6		---		---		100.0	
**2023**									
30	YES	0	30	0	30	0	30	28	2
93	NO	19	74	5	88	0	93	55	38
**E (%)**		100.0		100.0		---		-57.8	

Analyses were conducted in wild boar serum samples collected between 2018 and 2023 for serosurveillance (n = 382 for IV treated and n = 805 for untreated controls) as disclosed in in the manuscript and Supplementary Table 3. The E refers to reduction in pathogen prevalence.

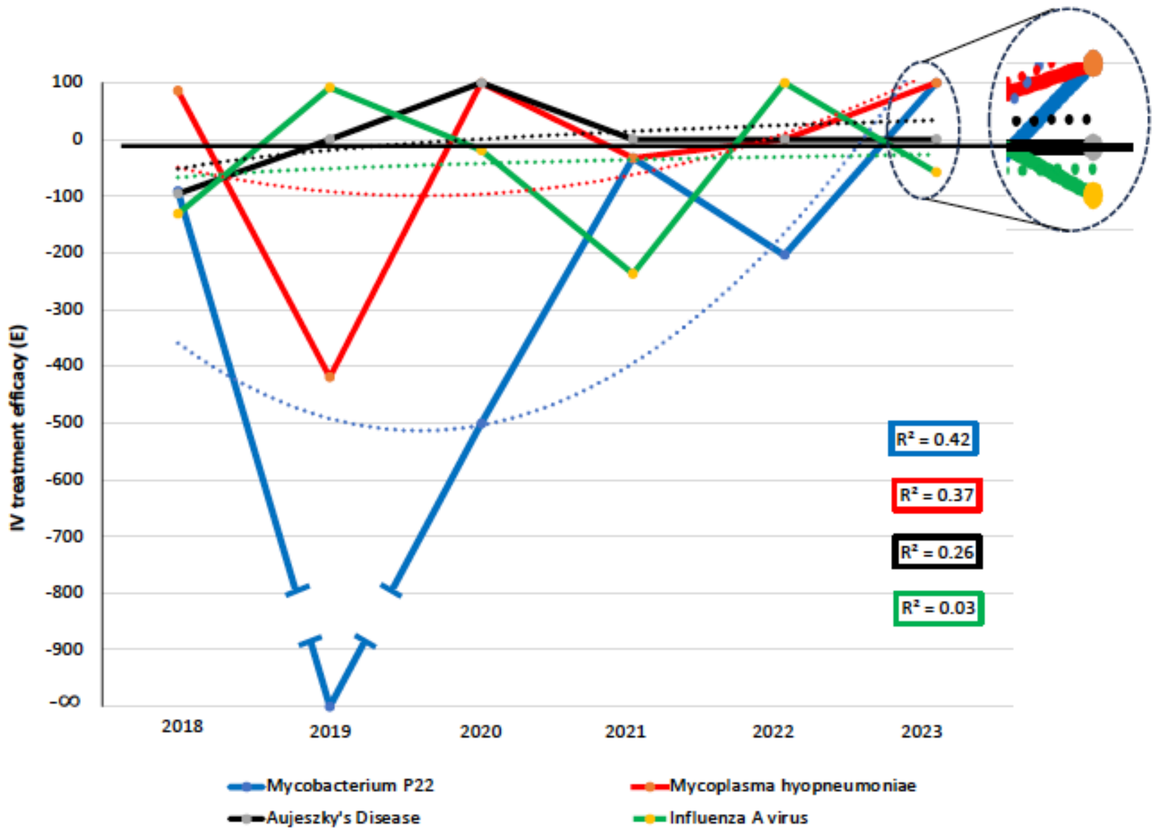

Trendline of IV treatment reduction on pathogen prevalence (E) over time. Polynomial trendlines and R squared values (R^2) for E and sampling year were calculated for each pathogen using Microsoft Excel software.

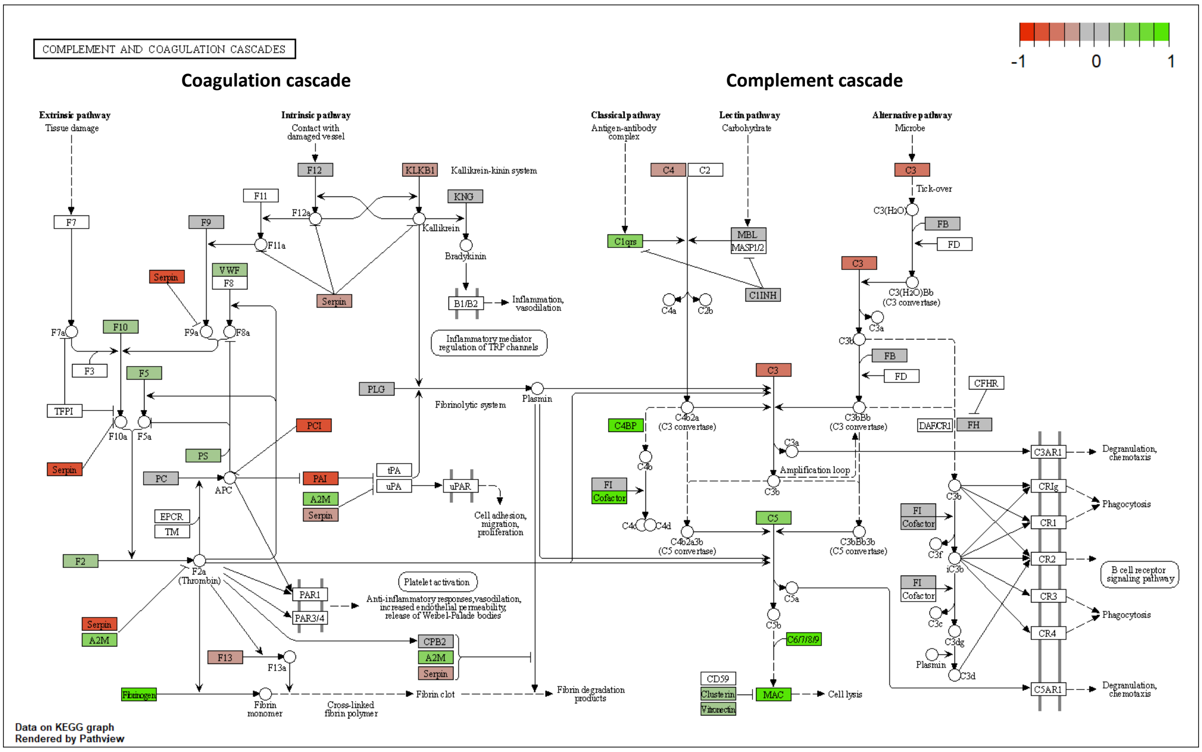

Supplementary Figure 1. Complement and coagulation cascades pathways. Results from KEGG database rendering involved activated (red) and suppressed (green) proteins from treated wild boar that were identified with SWATH-MS proteomics analysis. Created with *Pathview* function and package.
